# Nerve Echogenicity in Polyneuropathies of Various Etiologies—Results of a Retrospective Semi-Automatic Analysis of High-Resolution Ultrasound Images

**DOI:** 10.3390/diagnostics12061341

**Published:** 2022-05-28

**Authors:** Anke Erdmann, Jeremias Motte, Jil Brünger, Thomas Grüter, Ralf Gold, Kalliopi Pitarokoili, Anna Lena Fisse

**Affiliations:** 1 Department of Neurology, St. Josef-Hospital, Ruhr-University Bochum, 44791 Bochum, Germany; jeremias.motte@rub.de (J.M.); jil.bruenger@rub.de (J.B.); thomas.grueter@rub.de (T.G.); ralf.gold@rub.de (R.G.); kalliopi.pitarokoili@rub.de (K.P.); anna.fisse@rub.de (A.L.F.); 2 Immune-Mediated Neuropathies Biobank (INHIBIT), Ruhr-University Bochum, 44801 Bochum, Germany

**Keywords:** echogenicity, echo intensity, ultrasound, chronic inflammatory demyelinating polyneuropathy, critical illness polyneuropathy, chemotherapy-induced polyneuropathy

## Abstract

Echogenicity of peripheral nerves in high-resolution ultrasound (HRUS) provides insight into the structural damage of peripheral nerves in various polyneuropathies. The aim of this study was to compare nerve echogenicity in different primarily axonal or demyelinating polyneuropathies to examine the significance of this parameter. Performing semi-automated echogenicity analysis and applying Image J, we retrospectively used HRUS images of 19 patients with critical illness polyneuropathy (CIP), and 27 patients with chemotherapy-induced polyneuropathy (CIN) and compared them to 20 patients with chronic inflammatory demyelinating polyneuropathy (CIDP). The fraction of black representing echogenicity was measured after converting the images into black and white. The nerves of patients with progressive CIDP significantly differed from the hyperechogenic nerves of patients with other polyneuropathies at the following sites: the median nerve at the forearm (*p* < 0.001), the median nerve at the upper arm (*p* < 0.004), and the ulnar nerve at the upper arm (*p* < 0.001). The other polyneuropathies showed no notable differences. Altogether, the comparison of echogenicity between different polyneuropathies supports the assumption that there are differences depending on the genesis of the structural nerve damage. However, these differences are slight, and cannot be used to show clear differences between each polyneuropathy form.

## 1. Introduction

High-resolution nerve ultrasound (HRUS) has been presented in current studies as a valuable and promising diagnostic tool in various polyneuropathies [1,2,3,4,5]. Besides the nerve cross-sectional area (CSA), echogenicity was described as a parameter to depict the structural condition of nerves. According to Härtig et al. [6], hypoechogenic nerves in patients with chronic inflammatory demyelinating polyneuropathy (CIDP) reflect oedema and demyelination, whereas hyperechogenic nerves represent axonal damage with fibrous-scarring remodeling. In contrast, Puma et al. [7] described increased inflammatory infiltrates in hyperechogenic nerves. Furthermore, echogenicity was described as highly variable even in healthy individuals [4]. Therefore, the importance of nerve echogenicity in the evaluation of polyneuropathies is unclear.

Different methods for measuring nerve echogenicity have been used up to now: the measurement of the mean gray value [8], of the fraction of black [4], as well as qualitative patterns [6]. None of these have been used in polyneuropathies of various etiologies to understand the significance of alterations of echogenicity.

To shed more light on the diagnostic value of nerve echogenicity, we analyzed the echogenicity by the fraction of black in the ultrasounds of three different polyneuropathies: CIDP, critical illness polyneuropathy (CIP), and chemotherapy-induced neuropathy (CIN). Simplified, we chose CIDP as an inflammatory demyelinating disease on the one hand, while CIP and CIN were used to represent axonal polyneuropathies. The aim of the study was to elaborate on whether nerve echogenicity reflects different etiology of polyneuropathies.

## 2. Materials and Methods

The ethics committee of the Ruhr University in Bochum, Germany, approved our study protocol, and all patients signed informed consents (North-Rhine Westphalia Ethic Committee No 4905–14 for CIN, vote no. 18-6407 for CIDP and vote no. 16-5994 for CIP).

Inclusion criteria were: Clinically conclusive diagnosis of CIP, CIN or CIDP, age > 18 years, exclusion criteria were: Polyneuropathies of other etiology or multiple or unknown causes for polyneuropathy, pregnancy, other severe autoimmune diseases, underage subjects and subjects incapable of giving consent. Diagnosis of CIDP was made according to the EFNS PNS 2010 criteria [9].

### 2.1. Nerve Ultrasound

The peripheral nerves were examined at the following locations:

– Median nerve on the forearm, 10 cm above the retinaculum flexorum;
– Median nerve on the upper arm, the proximal end of the distal third between the elbow and the axilla;
– Ulnar nerve on the forearm, 10 cm proximal to the Guyon’s canal;
– Ulnar nerve on the upper arm, the proximal end of the distal third between the elbow and axilla;
– Radial nerve on the upper arm in the radial sulcus;
– Fibular nerve in the popliteal fossa;
– Tibial nerve at the medial malleolus.

All ultrasound examinations were performed with an AplioR XG ultrasound system (Toshiba Medicals, Tochigi, Japan) or an Affiniti 70G ultrasound system (Philips, Hamburg, Germany). In this regard, all investigators were certified neurologists with at least two years of experience in nerve and muscle ultrasound. Ultrasound was performed as previously described [4,10,11]. Importantly, all settings except depth and focus were kept constant and anisotropy was avoided by positioning the patient in a neutral position and placing the ultrasound probe only with its own weight and at a 90° angle to the nerve.

CSA data, which are not part of this manuscript, as well as echogenicity for patients with CIDP and CIP, were reported in previous manuscripts with different aims [1,11] but the analysis of echogenicity of CIN and the comparison of the echogenicity of all three polyneuropathies is new in this manuscript. For the sake of completeness, a synopsis of the CSA data can be found in Supplementary Table S1.

### 2.2. Image Selection

All patients were examined using HRUS longitudinally in the disease course several times. To show the greatest possible differences in echogenicity between the different polyneuropathies, we analyzed the ultrasound images of the examination time point at which the polyneuropathy was most pronounced clinically and in nerve conduction studies. For each group, this image selection was performed as follows:

#### 2.2.1. CIP Group

Patients with CIP were previously described in a paper on clinical, electrophysiologic, and sonographic characteristics of CIP [1]. They underwent clinical, sonographic, and electrophysiological examinations every 7th day after admission to the intensive care unit until discharge from the hospital or death. The severity of the polyneuropathy in this study was evaluated using the CIP severity score calculated from the electrophysiological examinations as previously described [1]. This score showed the peak of the disease on day 7 after inclusion in the study, so the HRUS examination performed on the same day was used for our echogenicity analysis.

#### 2.2.2. CIN Group

Patients with CIN received platinum-based chemotherapy in the setting of malignant disease, with the platinum dose adjusted according to tolerability. All patients underwent multiple clinical, sonographic, and electrophysiological examinations. Thirteen were published in a previous study on the clinical, electrophysiologic, and sonographic characteristics of oxaliplatin-induced polyneuropathy [12]. We calculated the cumulative dose of platinum-based chemotherapeutic agents and evaluated the nerve conduction studies in the period of the maximal platinum dose in terms of the amplitude reduction of sensitive nerve action potentials (sNAP) and the muscle sum action potentials (cMAP) reflecting axonal damage. The ultrasound images at the time of the most pathological nerve conduction findings were used for echogenicity analysis.

#### 2.2.3. CIDP Group

Patients with CIDP were examined every 6th month for a period of 34 months in a previous study on the long-term changes of the intranerve cross-sectional area variability in CIDP [13]. The data on the echogenicity of these patients were already published as described above [4,11] and were used in our study for comparison of polyneuropathy groups. Patients were divided into two groups according to the Overall Disability Sum Sores (ODSS). First, patients who showed a stable or regressive disease course (*n* = 12) and second, patients who showed a progressive course (*n* = 8), defined as an increase of the ODSS [14] ≥1 during the study period. All examinations collected from the patients during the study period were analyzed.

### 2.3. Evaluation of Echogenicity

**Figure 1:**
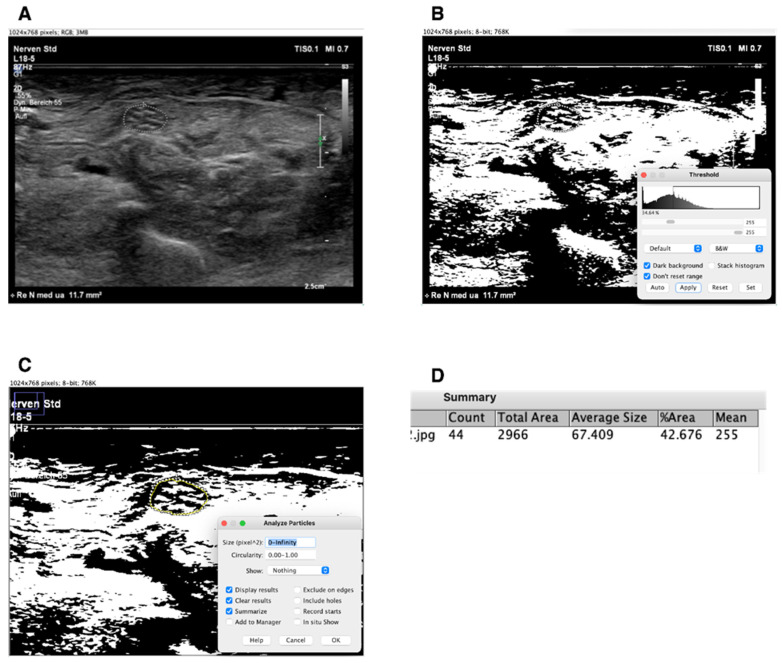
Analyzing ultrasound pictures in Image J. (**A**) = median nerve (position lower arm) in a raw ultrasound picture; (**B**) = the same image converted to 8-bit and modifying threshold to black and white. Automatic threshold function is presented in the lower right corner of the picture in the small window; (**C**) = now the median nerve in the image is bordered by freehand tracing; settings for analysis of the traced particles are shown on the window on the bottoms right; (**D**) = finished analysis of the traced particle, the result shows the fraction of white in %. The fraction of black is the difference from 100 minus the fraction of white.

Regarding the evaluation of echogenicity, semi-automatic image analysis was performed using ImageJ, as also described previously in Fisse et al. [11], and the fraction of black was quantified. For this purpose, the nerves were marked during the examination, manually outlined, and then converted to 8-bit. Each pixel appears in a range between 0 (black) and 255 (white). Subsequently, an automatic histogram segments the grey levels of the image into black and white pixels using the threshold function. Finally, the hyperechogenic fraction, i.e., the white pixels, can be calculated proportionally as a percentage. The difference to 100 represents the fraction of black. An example is shown in Figure 1.

### 2.4. Statistics

Statistical analysis was performed using Microsoft Excel for Mac version 16.25 (Microsoft, Redmond, WA, USA) to generate boxplots and create graphs. Furthermore, IBM SPSS Statistics 25 (IBM Corporation, Armonk, NY, USA) was used for group comparisons. Absolute data are presented as mean ± SD or as the median with range, lower and upper quartile. Differences between groups were tested by the Mann–Whitney U test, *t*-test or Chi-squared test, as applicable. The statistically significant threshold was set at a *p*-value of <0.05. We calculated the mean values and standard deviations to perform a one-factor analysis of variance (ANOVA) analysis. At the significant sites, we conduct Bonferroni post hoc testing.

## 3. Results

### 3.1. Patients

A total of 66 patients were included in this study. Among them, 19 patients had CIP, and 27 patients had CIN and 20 patients had CIDP (progressive disease course *n* = 8, stable or regressive disease course *n* = 12). The mean age (±SD) of patients with CIP was 65 (±13) years, that of patients with CIN was 64 (±8) years and that of patients with CIDP was 53 (±14) years. All CIDP patients had typical proximal and distal sensorimotor polyneuropathy and fulfilled the 2010 diagnostic EFNS/PNS criteria [9]. All CIN patients had typical distal sensorimotor polyneuropathy after the beginning of chemotherapy. CIP patients either had intensive care unit acquired weakness plus signs of polyneuropathy in nerve conduction studies or, if they did not reach a sufficient level of consciousness for a reliable muscular assessment during intensive care unit treatment, proof of sensorimotor polyneuropathy in nerve conduction studies. Baseline characteristics can be found in Table 1. Supplementary Table S2 gives an overview of each included patient with a corresponding diagnosis.

### 3.2. Comparison of Nerve Echogenicity of the Different Polyneuropathies

**Figure 2:**
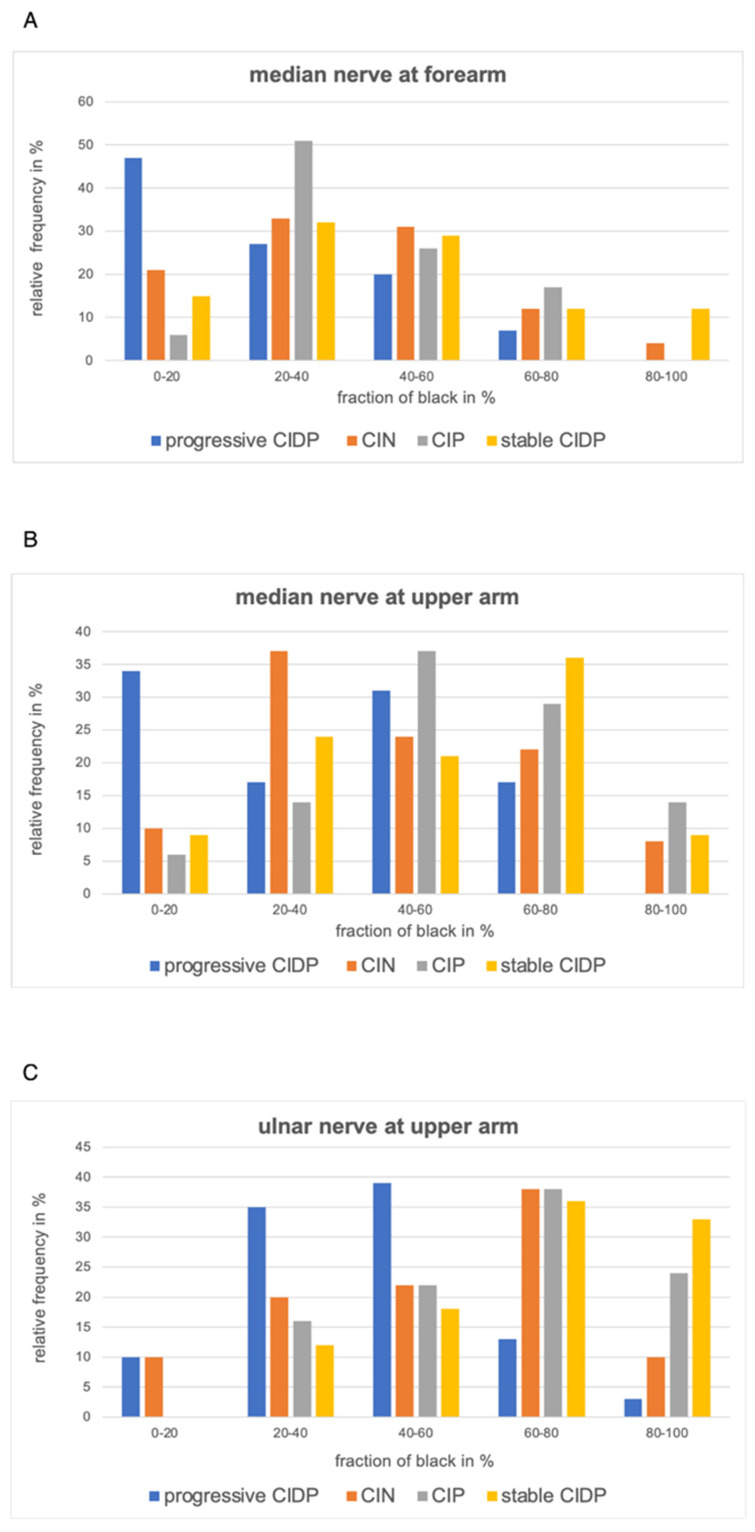
Relative frequency distribution of the fraction of black of the significant locations, (**A**) = median nerve in forearm, (**B**) = median nerve in upper arm, (**C**) = ulnar nerve in upper arm.

**Figure 3:**
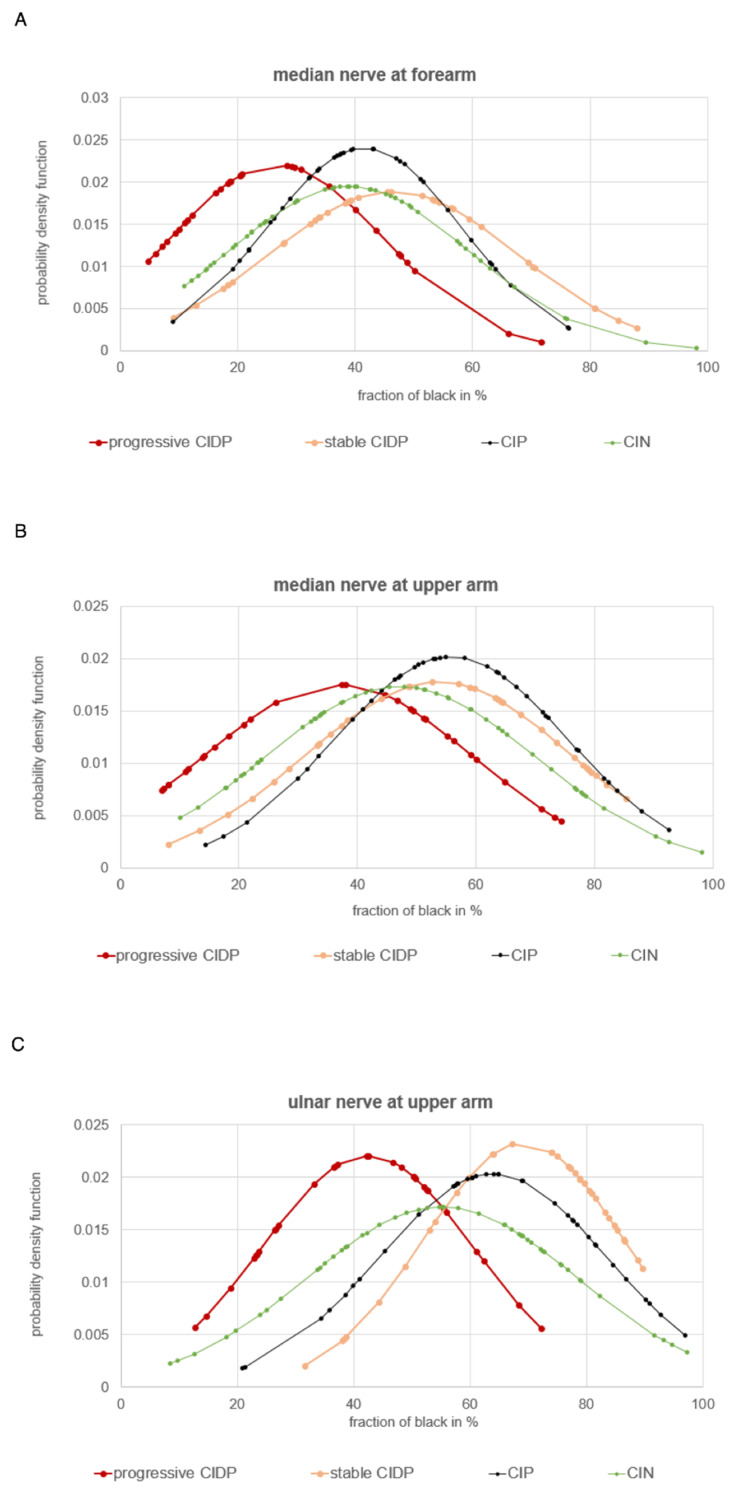
Distribution of echogenicity of the significant examination sites of patients with CIDP (progressive disease course = red, *n* = 8 and stable/regressive disease course = orange, *n* = 12) in comparison to patients with CIP (black, *n* = 19) and CIN (green, *n* = 27). (**A**): median nerve in forearm, (**B**): median nerve in upper arm, (**C**): ulnar nerve in upper arm.

**Figure 4:**
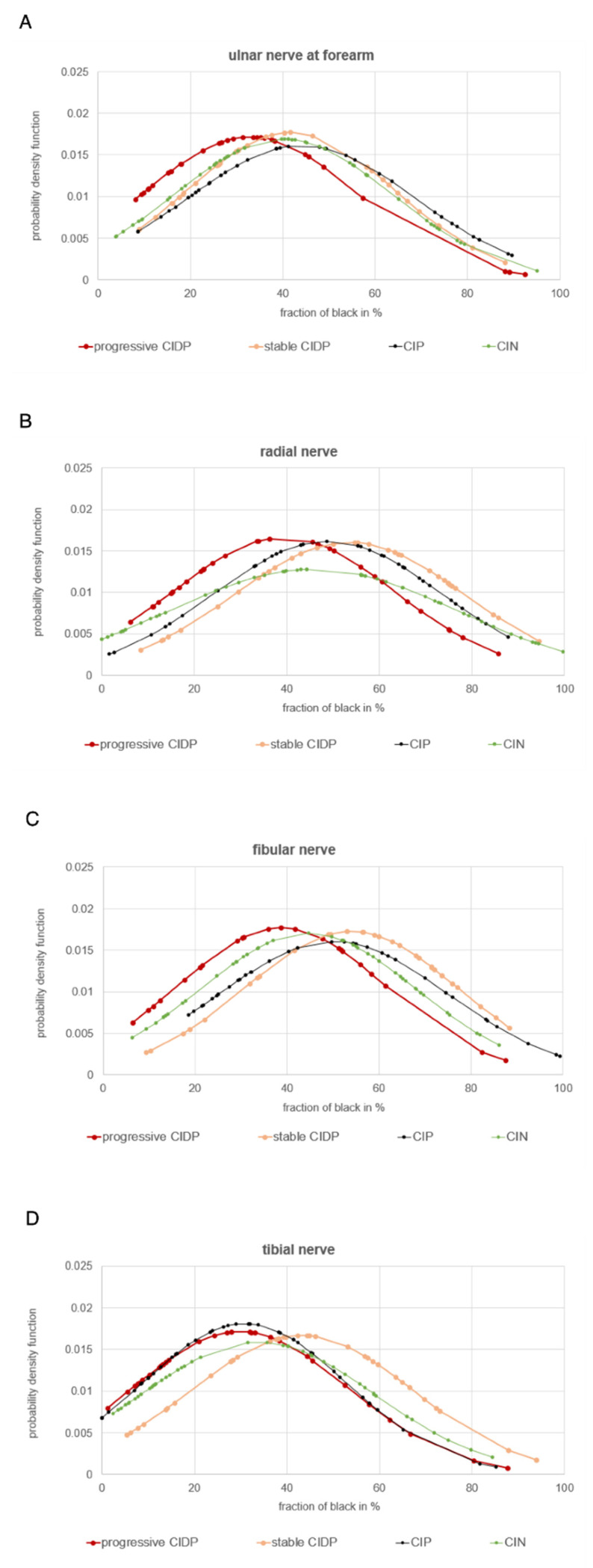
Distribution of echogenicity of the non-significant examination sites of patients with CIDP (progressive disease course = red, *n* = 8 and stable/regressive disease course = orange, *n* = 12) in comparison to patients with CIP (black, *n* = 19) and CIN (green, *n* = 27). (**A**) = ulnar nerve in forearm, (**B**) = radial nerve, (**C**) = fibular nerve, (**D**) = tibial nerve.

Considering all measured sites together, patients with progressive CIDP had an average fraction of black of 35.1% with a standard deviation of ±22.1, while patients with CIN had 43.7% (±25.1), patients with CIP had 47.9% (±23.8), and patients with stable CIDP had 51.6% (±23.6). Thus, nerve ultrasound images of patients with CIDP with a progressive disease course show more echo intense nerves than patients in the other groups. The echogenicity of patients with stable CIDP, patients with CIP, and patients with CIN appear overall similar; on a closer look, patients with CIN seem to have a slightly lower fraction of black than patients with stable CIDP and CIP. These findings are most evident in the nerves of the upper extremity, though with a high standard deviation: The median nerve at the forearm in patients with progressive CIDP has an average fraction of black of 26.7% (±18.1), whereas patients with CIN, CIP and stable CIDP show values of 38.9% (±20.5), 41.6% (±16.6), and 46.5% (±21.1), respectively. Similar differences occur at the median nerve in the upper arm, with a fraction of black of 36.5% (±22.7) in patients with progressive CIDP, 47.2% (±23) in patients with CIN, 53.7% (±22.5) in patients with stable CIDP, and 55.8% (±19.8) in patients with CIP. Lastly, the ulnar nerve at the upper arm in patients with progressive CIDP showed a fraction of black of 42.4% (±18.1), whereas patients with CIN had a fraction of black of 55.2% (±23.2), patients with CIP had 64% (±19.6) black, and patients with stable CIDP had 69.2% (±17.1) black. The overview of the frequency distribution can be found in Figure 2, Figure 3 and Figure 4, as well as the exact values in Table 2.

Accordingly, in the statistical analysis, the performed one-factor ANOVA analysis and the Bonferroni-corrected post hoc test specified significant differences at the locations of the arm nerves just described: median nerve in the forearm (*p* < 0.001), the median nerve in the upper arm (*p* 0.003) and ulnar nerve at upper arm (*p* < 0.001) with differences between patients with progressive CIDP and with stable CIDP as well as patients with progressive CIDP and with CIP and CIN. Of the three locations, the ulnar nerve at the upper arm shows the largest mean difference between the means of the groups: The upper arm ulnar nerve of patients with progressive CIDP show an average of 12.8% lower fraction of black compared to the patients with CIN, of 21.55% lower compared to patients with CIP and of 26.8% lower compared to patients with stable CIDP. Furthermore, there is also a significant difference between patients with CIN and patients with stable CIDP at the ulnar nerve in the upper arm. Here, the mean fraction of black of patients with CIN is 14% lower than that of patients with stable CIDP. There are no significant mean differences between the images of patients with CIN and patients with CIP, or patients with CIP and patients with stable CIDP at any site. The detailed results of the Bonferroni post hoc test can be found in Table 3. The echogenicity of the nerves of the lower limb did not differ significantly between the groups.

Looking at the distribution of the fraction of black in Figure 3 and Figure 4, it is noticeable that all four curves have a wide range of values that overlap with the other curves, which is also evident in the quite high standard deviations.

## 4. Discussion

We presented a retrospective study comparing nerve echogenicity of different polyneuropathies to gain a better understanding of the alterations of echogenicity depending on the etiology of a neuropathy (inflammatory demyelinating versus axonal). Taken together, we were able to show the following points in our study:

– The patients with progressive CIDP show hyperechogenic nerves compared not only to patients with stable CIDP, which was published before [11] but also to the other polyneuropathies, CIP and CIN. Patients with progressive CIDP presumably showed secondary axonal damage with fibrotic remodeling associated with a lower response to therapy [13]. This supports the thesis of hyperechoic nerves occurring in axonal fibrous remodeling.
– The echogenicity of patients with stable CIDP, CIP and CIN did not show much difference. On a closer look, patients with CIN seem to have a slightly lower fraction of black than patients with stable CIDP and CIP. This could support the thesis that in the context of this comparative study, patients with CIN had the most chronic axonal neuropathy, while patients with CIP were examined in the acute phase characterized by inflammation [1], such as in stable CIDP. However, this is an aspect of patient selection of this study, not a consistent characteristic of the diseases. Therefore, based on our study, it is not possible to distinguish between the individual polyneuropathies by analysis of echogenicity.
– Each of the four polyneuropathies shows a wide range of echogenicity on its own, overlapping with the other polyneuropathies; therefore, the polyneuropathies groups cannot be defined or diagnosed by a specific range of echogenicity.

As a conclusion of our results, consideration of echogenicity could have potential use in the follow-up of patients with polyneuropathy: If further studies confirm that hyperechogenic nerves correspond to fibrous remodeling, potential therapeutic approaches, such as neuro-regenerative and neuro-protective approaches and rehabilitation could be used in patients with hyperechogenic nerves, while in patients with hypoechogenic nerves, anti-inflammatory treatment for inflammatory neuropathies could rather be beneficial.

Looking at the current literature, only very few studies reported a quantitative analysis of the echogenicity of peripheral nerves in CIDP, CIN and CIP.

– Literature on CIDP: Padua et al. [15] reported echogenicity changes in patients with CIDP in 2014, describing hypoechogenic, hyperechogenic, and mixed nerves. He firstly postulated that hypoechogenic nerves indicate inflammation and oedema, whereas hyperechogenic nerves indicate irreversible axonal damage. This was supported by further studies, showing that hyperechoic nerves in CIDP were associated with a worse response to therapy [6] and progressive disease course [11]. However, these studies used qualitative scores for analyzing echogenicity, not a quantitative measurement of a fraction of black or gray value. It is worth noting that the Heckmatt score has been established in the field of muscle ultrasound for the assessment of echogenicity changes [16]. However, for nerve ultrasound to date, there is no standard evaluation method for echogenicity.
– Literature on CIP: To our knowledge, there is only our own previous study reporting on nerve echogenicity in the acute phase of the disease [1]. Looking at the pathogenesis of CIP, which is a primary axonal polyneuropathy, also inflammatory processes play a key role, especially in the acute phase. Numerous inflammatory mediators, such as overexpression of E-selectin and increased cytokine production, which may lead to endoneural oedema due to increased vascular permeability, are of importance [17]. This could be the reason that nerves may present as more hypoechogenic compared to progressive CIDP and stable CIDP in HRUS.
– Literature on CIN: Regarding the CIN literature on echogenicity, it is not very productive for our research, as well. To date, only a few studies present nerve ultrasound changes in patients receiving platinum-based chemotherapy. Those who do, primarily focus on the parameter of CSA in their study [12,18] and do not report on echogenicity. Increased cytokine and chemokine production, as well as activation of glial cells, which subsequently attract and activate immune cells, can also be found in CIN, but CIN is generally not considered to be inflammatory [19]. Therefore, this supports that CIN exhibits similar or maybe slightly hyperechogenic nerves than does CIP and stable CIDP remodeling.

## 5. Limitations

The major limitation of this study is the methodological design. The study was retrospective and used HRUS images, which were originally intended for size measurement. Furthermore, images were acquired only once. For further studies, more frequent recording with a generation of an average value for the fraction of black seems necessary to show the reliability of the method; moreover, the determined time of the examination in which each polyneuropathy was most pronounced. As nerve echogenicity may change during the disease, the precise determination of the appropriate HRUS examination is of particular importance. This is critical, as the longitudinal characteristics of the diseases are inherently different and difficult to compare. Therefore, it is difficult to accurately correlate the disease courses so that each group can be examined at the identical disease state of the severity of the neuropathy. Furthermore, our patients had different disease duration. This fact not only plays a role in the chosen timing of HRUS examination but also directly affects echogenicity, therefore, confounding may occur. Another point to consider is certainly the technical limitations of the ultrasound system to differentiate the grayscale. The echogenicity depends strongly on the settings of the ultrasound system. Even if we kept all settings constant, we determined the grey levels with a separate program, which does not make a reference to clear determinants, such as vessels or bones.

## 6. Conclusions

Summarized, the comparison of nerve echogenicity of different polyneuropathies by semi-automatic image analysis shows that echogenicity probably cannot distinguish between demyelinating inflammatory and axonal polyneuropathies in general, however, it might be possible to gain limited insights into the pathogenesis of polyneuropathy. Especially for patients with progressive CIDP, it seems to reveal hyperechogenic nerves compared to the other polyneuropathies, probably reflecting fibrous remodeling.

Prospective studies are needed to further evaluate nerve echogenicity and offer an interesting outlook for the future.

## Figures and Tables

**Table 1:** Baseline characteristics of all groups.

	CIDP (Progressive) (*n* = 8)	CIDP (Stable) (*n* = 12)	CIP (*n* = 19)	CIN (*n* = 27)
Age (mean ± SD)	56 ± 12 years	52 ± 16 years	65 ± 13 years	64 ± 8 years
women (%)	38	42	32	37
disease duration until inclusion (median (IQR))	5 years (4)	3 years (4)	7 days (5)	2 months (2)
overall disability sum score at inclusion (median (IQR))	4 (1)	4 (1)		
duration of intensive care unit treatment in days (median (IQR))			23 days (18)	
chemotherapy dose (mean ± SD)				1565 ± 842 mg Oxaliplatin (*n* = 24) or 750 mg Carboplatin (*n* = 1) or 798 ± 31 mg Cisplatin (*n* = 2)

**Table 2:** Mean fraction of black of all groups.

	Progressive CIDP	CIN	CIP	Stable CIDP
**all regions**	35.1 ± 22.1	43.7 ± 25.1	47.9 ± 23.8	51.6 ± 23.6
**Median nerve** **lower arm**	26.7 ± 18.1	38.9 ± 20.5	41.6 ± 16.6	46.5 ± 21.1
**Median nerve** **upper arm**	36.5 ± 22.7	47.2 ± 23	55.8 ± 19.8	53.7 ± 22.5
**Ulnar nerve** **lower arm**	34 ± 23.3	40.1 ± 23.6	44.1 ± 24.8	41.8 ± 22.5
**Ulnar nerve** **upper arm**	42.4 ± 18.1	55.2 ± 23.2	64 ± 19.6	69.2 ± 17.1
**Radial nerve**	39.3 ± 24	45.6 ± 31.2	48.8 ± 24.7	53.5 ± 24.9
**Fibular nerve**	38.8 ± 22.6	44.6 ± 23.5	50.1 ± 24.9	53.9 ± 23.1
**Tibial nerve**	29.9 ± 23.3	33.7 ± 25.1	30.9 ± 22.1	43.2 ± 23.9

Data represent mean ± standard deviation.

**Table 3:** Significant and non-significant differences in the Bonferroni-adjusted post hoc analysis.

	Median Nerve Lower Arm	Median Nerve Upper Arm	Ulnar Nerve Upper Arm
**CIDP progressive disease course** vs. **CIDP regressive/stable disease course**	−19.8 * (*p* < 0.001)	−16.92 * (*p =* 0.018)	−26.8 * (*p* < 0.001)
**CIDP progressive disease course** vs. **CIN**	−12.29 * (*p* 0.038)	−10.45 * (*p* 0.0262)	−12.8 * (*p* 0.037)
**CIDP progressive disease course** vs. **CIP**	−14.91 * (*p* 0.014)	−19.09 * (*p* 0.004)	−21.55 * (*p* < 0.001)
**CIDP regressive/stable disease course** vs. **CIN**	7.51 (*p* 0.482)	6.47 (*p* 1.000)	14 * (*p* 0.014)
**CIDP regressive/stable disease course** vs. **CIP**	4.89 (*p* 1.000)	−2.17 (*p* 1.000)	5.26 (*p* 1.000)
**CIP** vs. **CIN**	2.61 (*p* 1.000)	8.64 (*p* 0.43)	8.75 (*p* 0.281)

Mean difference in percent; in parentheses, significance. * the mean difference between the groups is significant at the 0.05 level.

## Data Availability

Data collected from this study are available by emailing jeremias.motte@rub.de.

## Supplementary Materials

The following supporting information can be downloaded at: https://www.mdpi.com/article/10.3390/diagnostics12061341/s1, Table S1: CSA data of all groups; Table S2: Detailed Data of all patients.
