# Supplementary material for: Nerve Echogenicity in Polyneuropathies of Various Etiologies—Results of a Retrospective Semi-Automatic Analysis of High-Resolution Ultrasound Images
Source: Diagnostics (Basel). 2022 May 28;12(6):1341. doi: 10.3390/diagnostics12061341 (PMC9221766; doi:10.3390/diagnostics12061341)
Supplement: Supplementary file 1 [file diagnostics-12-01341-s001.zip › diagnostics-1690683-supplementary.pdf]

Supplementary

Table S1. CSA data of all groups. Data represents mean (mm²) ± standard deviation.

	CIDP (progressive)	CIDP (stable)	CIP	CIN
**Median nerve lower arm**	8.6 ± 2.9	11.1 ± 3.9	8 ± 2.7	8 ± 1.94
**Median nerve upper arm**	12.3 ± 5.1	14.8 ± 7.1	8.9 ± 2.5	8.9 ± 2.6
**Ulnar nerve lower arm**	6.2 ± 2.5	7.3 ± 2.9	5.7 ± 1.3	5.7 ± 1.3
**Ulnar nerve upper arm**	7.9 ± 3	10.3 ± 4.8	6.4 ± 2.2	7.3 ± 2.59
**Radial nerve**	5.9 ± 3.2	7.5 ± 4.3	4.6 ± 1.6	5.06 ± 2.12
**Fibular nerve**	12.4 ± 7.1	13.9 ± 9.6	6.6 ± 1.8	8.9 ± 3.17
**Tibial nerve**	10.5 ± 4.1	13.7 ± 6.8	7.5 ± 3.9	10.7 ± 3.5

Table S2. Detailed Data of all patients.

ID	Age, gender	Diagnosis	Duration of ICU treatment in days	Chemo-therapy dose	ODSS at inclusion
**1**	44, f	CIDP, progressive			6
**2**	58, m	CIDP, progressive			4
**3**	45, m	CIDP, progressive			3
**4**	59, m	CIDP, progressive			5
**5**	75, m	CIDP, progressive			4
**6**	53, m	CIDP, progressive			3
**7**	43, m	CIDP, progressive			1
**8**	67, m	CIDP, progressive			2
**9**	57, f	CIDP, stable			3
**10**	72, m	CIDP, stable			6
**11**	48, m	CIDP, stable			4
**12**	44, f	CIDP, stable			6
**13**	31, m	CIDP, stable			5
**14**	18, m	CIDP, stable			4
**15**	53, m	CIDP, stable			4
**16**	47, f	CIDP, stable			11
**17**	59, f	CIDP, stable			3
**18**	60, m	CIDP, stable			4
**19**	78, m	CIDP, stable			5
**20**	57, f	CIDP, stable			3
**21**	70, m	CIN, esophageal carcinoma		1170 mg oxaliplatin	
**22**	54, m	CIN, gastric adeno-CUP		750 mg carboplatin	
**23**	57, f	CIN, gastric carcinoma		1785 mg oxaliplatin	
**24**	76, f	CIN, gastric carcinoma		1272 mg oxaliplatin	
**25**	68, f	CIN, gastric carcinoma		3840 mg oxaliplatin	

**26**	57, m	CIN, gastric carcinoma		1154 mg oxaliplatin
**27**	74, m	CIN, non-small cell lung cancer		820 mg cisplatin
**28**	44, m	CIN, non-small cell lung cancer		776 mg cisplatin
**29**	66, f	CIN, pancreatic cancer		1452 mg oxaliplatin
**30**	64, m	CIN, pancreatic cancer		3061 mg oxaliplatin
**31**	60, m	CIN, pancreatic carcinoma		1596 mg oxaliplatin
**32**	57, m	CIN, pancreatic carcinoma		1848 mg oxaliplatin
**33**	74, f	CIN, pancreatic carcinoma		980 mg oxaliplatin
**34**	61, m	CIN, pancreatic carcinoma		1980 mg oxaliplatin
**35**	60, f	CIN, pancreatic carcinoma		1150 mg oxaliplatin
**36**	53, m	CIN, pancreatic carcinoma		1360 mg oxaliplatin
**37**	56, m	CIN, pancreatic carcinoma		2226 mg oxaliplatin
**38**	63, m	CIN, pancreatic carcinoma		1670 mg oxaliplatin
**39**	63, m	CIN, pancreatic carcinoma		1979 mg oxaliplatin
**40**	67, f	CIN, pancreatic carcinoma		2496 mg oxaliplatin
**41**	74, f	CIN, pancreatic carcinoma		1212 mg oxaliplatin
**42**	62, f	CIN, pancreatic carcinoma, HNPCC		1304 mg oxaliplatin
**43**	80, m	CIN, rectal carcinoma		162 mg oxaliplatin
**44**	68, m	CIN, rectal carcinoma		153 mg oxaliplatin
**45**	73, m	CIN, rectal carcinoma		1360 mg oxaliplatin
**46**	64, m	CIN, small cell lung cancer		438 mg oxaliplatin
**47**	66, f	CIN, small cell lung cancer		1920 mg oxaliplatin
**48**	61, m	CIP, acute exacerbation of COPD, pneumonia	10	
**49**	58, f	CIP, cardiopulmonary resuscitation	46	
**50**	67, m	CIP, cardiopulmonary resuscitation, cerebral edema	10	
**51**	60, m	CIP, cerebral infaction	48	
**52**	82, m	CIP, epileptic seizure with cardiopulmonary resuscitation	16	

**53**	33, f	CIP, meningoencephalitis	21
**54**	79, f	CIP, NSTEMI, asystole	10
**55**	64, m	CIP, pneumococcal sepsis	37
**56**	69, m	CIP, pneumococcal sepsis	33
**57**	76, m	CIP, pneumococcal sepsis, acute hypercapnic respiratory failure	35
**58**	74, m	CIP, pneumonia, acute kidney injury	30
**59**	81, m	CIP, pneumonia, acute respiratory distress syndrome	30
**60**	51, f	CIP, pneumonia, choreatiform movement disorder with dysphagia	23
**61**	63, f	CIP, seizures with lactic acidosis, hyperprolinemia	69
**62**	53, m	CIP, STEMI, cardiogenic shock, cardiopulmonary resuscitation	13
**63**	55, m	CIP, STEMI, cardiogenic shock, cardiopulmonary resuscitation	61
**64**	62, f	CIP, urosepsis	21
**65**	62, m	CIP, ventricular fibrillation	12
**66**	82, m	CIP, ventricular fibrillation	22
